# Rapid Documentation of Possible Semen Stains for Forensic DNA Profiling

**DOI:** 10.3390/genes16091073

**Published:** 2025-09-12

**Authors:** Zhonghui Thong, Audrey Ping Jue Wee, Baoqiang Heng, Christopher Kiu Choong Syn

**Affiliations:** DNA Profiling Laboratory, Biology Division, Health Sciences Authority, 11 Outram Road, Singapore 169078, Singapore; audrey_wee@hsa.gov.sg (A.P.J.W.); heng_baoqiang@hsa.gov.sg (B.H.); christopher_syn@hsa.gov.sg (C.K.C.S.)

**Keywords:** acid phosphatase, AP-positive, transfer paper, semen, documentation

## Abstract

The acid phosphatase (AP) test is widely utilised in forensic biology laboratories to examine for the presence of semen on crime evidence. If semen is present, the AP-positive areas are marked on the exhibit to indicate the precise location of the semen stain. However, documenting AP-positive areas with a crayon is time-consuming and laborious. In this proof-of-concept study, we evaluated the use of Saral Wax-Free Transfer Taper (TP) as an alternative tool for tracing the boundaries of AP-positive areas. We demonstrated that the TP pigment did not inhibit PCR amplification, as indicated by consistent internal PCR control (IPC) C_T_ values during real-time DNA quantification. While a reduction in DNA yield was observed under stress-test conditions, where TP pigment was intentionally included in the samples, complete STR profiles were still recovered with no allele dropout. Importantly, the documenting time for AP mapping was reduced by approximately five-fold with TP compared to crayon, underscoring its potential to enhance efficiency in forensic laboratory workflows.

## 1. Introduction

Acid phosphatase (AP) is a water-soluble enzyme secreted by the prostate gland and found in seminal fluid at concentrations that are at least 200 times higher than those in other bodily fluids [1,2,3]. Leveraging this property, forensic scientists have utilised the AP test for presumptive detection of semen since the 1950s [4,5]. Like other presumptive tests for bodily fluids such as blood and semen, the AP test can yield false-positive results [4,6,7,8,9]. This outcome is not unexpected, as AP is also present in certain vegetable juices and other bodily fluids [2,3,4,6,7,8,9]. To confirm semen detection after a positive AP result, forensic scientists can examine samples microscopically for spermatozoa and/or conduct immunoassays such as the Seratec^®^ PSA Semiquant test (Seratec, Göttingen, Germany) to detect prostate-specific antigen and/or the Rapid Stain Identification (RSID)^TM^-Semen test (Independent Forensics, Lombard, IL, USA) to detect semenogelin [10,11,12].

Despite its lack of specificity, the AP test has been widely adopted in the forensic community due to its rapid, sensitive, and straightforward methodology, making it especially useful for detecting semen stains in forensic casework. Additionally, the AP test is also more practical for screening large crime exhibits such as bedding compared to other presumptive tests for semen [3,4,6,9,13].

The AP test can be applied directly to the surfaces of exhibits such as cotton swabs, but more commonly, it is applied indirectly via a process known as the “press test” [3,4,9,12,13]. This process involves pressing moistened blotting or filter paper firmly onto the exhibit’s surface to transfer any semen present to the paper. The paper is then removed and sprayed with AP reagent, which results in a purplish colour in the presence of AP. Specifically, α-naphthyl phosphate is hydrolysed to α-naphthol, which then reacts with Brentamine Fast Blue to form a purple azo dye [3,4,5,6,7,8,9]. Strong purple colour development indicates a high level of AP activity, suggesting the presence of semen at the corresponding location on the exhibit. The dried filter paper can then be placed over the dried exhibit, and the AP stain is documented on the exhibit using a crayon. This process involves flipping the filter paper back and forth to accurately outline the purple-staining pattern, ensuring it aligns with its original location on the exhibit. Documenting both the paper and the exhibit is essential to enable accurate relocation of AP-positive areas for subsequent confirmatory tests, such as microscopy [3,4,9,13,14,15]. However, this crayon-based documentation process is time-consuming and laborious, particularly when there are multiple stains on a large exhibit.

The present study sought to explore a more practical approach towards documenting AP-positive areas. Saral Wax-Free Transfer Paper (TP) (Saral Paper Corp, Jersey City, NJ, USA), also referred to as graphite paper, has long been used in arts and crafts to transfer designs from one surface to another. Here, we evaluated its use as a tool to outline AP stain boundaries on exhibits. We further assessed whether TP impacts downstream DNA laboratory processes and compared its documenting time against the conventional crayon method.

## 2. Material and Methods

This study first involved assessing the effects of three different colour pigments (white, yellow and red) of Saral Wax-Free Transfer Paper (TP) on protein immunoassays and DNA processing, followed by an evaluation of TP for documenting AP-positive areas on mocked crime exhibits, consisting of a cotton T-shirt stained with semen.

### 2.1. Assessing the Effect of TP Pigment on Protein Immunoassays

Seven sections (~1 cm × 1 cm) of TP were prepared for each colour and placed into separate Eppendorf tubes for subsequent protein immunoassays, as described in Section 2.6.

### 2.2. Assessing the Effect of TP Pigment on DNA Processing

Seven sections (~1 cm × 1 cm) of TP were prepared for each colour and subjected to Maxwell (*n* = 2) and differential DNA extractions (*n* = 5), as described in Section 2.7.

### 2.3. Assessing the Effect of TP Pigment on Semen Detection via Protein Immunoassays

TP was placed with the pigment side facing down onto a cotton cloth. Firm pressure was applied using shading motions with a pen, causing the pigment from the TP to be transferred to the cotton surface, leaving visible colouration. For each coloured TP, seven cloth sections (~1 cm × 1 cm) were excised and spiked with 5.5 µL of diluted semen (Cat No. IRHUSMS5ML, Innovation Research, Novi, MI, USA) and then allowed to air-dry overnight prior to protein immunoassay testing.

### 2.4. Assessing the Effect of TP Pigment on DNA Processing from Semen Stains

For each coloured TP, seven cloth sections (~1 cm × 1 cm) coloured with TP pigment (similar to Section 2.3) were spiked with diluted semen, then subjected to Maxwell (*n* = 2) and differential DNA extractions (*n* = 5).

### 2.5. Documentation of AP-Positive Areas on Semen-Stained Cotton T-Shirt

Whatman Grade 1 Cellulose Chromatography Paper (Cat No. 3001-917, Cytiva, Marlborough, MA, USA) was moistened with ultrapure water and placed over the semen stains on the cotton T-shirt. A piece of dry chromatography paper was then placed over the moistened paper for support, and even pressure was applied over the area via a handheld roller. The moistened paper was allowed to air-dry in a fume hood. AP SPOT Test reagent (Lot No. 1969, SERI, Richmond, CA, USA) was sprayed onto the paper, and purple colouration indicated AP activity. Any spreading of the semen stain would have occurred during these wet application phases. The dried paper was placed back over the corresponding area on the dried T-shirt, and the AP-positive areas were documented using either a crayon or TP. For AP mapping using both crayon and TP, competent staff applied moderate, even pressure, consistent with the laboratory’s routine practice for AP documentation. Pressure was not quantitatively measured in this exploratory study but was guided by laboratory SOP to ensure visible markings without fabric damage. For TP documentation, tracing was performed using a standard stylus with a 2 mm tip. The SciPy library (version 1.12.0) was used to conduct a Wilcoxon signed-rank test. Effect size (Cohen’s *d*) was calculated by the mean difference divided by the pooled standard deviation.

### 2.6. Protein Immunoassays

Samples were incubated in 100 µL of RSID^TM^ Universal buffer on a thermomixer at 25 °C for 30 min with agitation at 1000 rpm. After centrifugation (to pellet sperm in semen-containing samples), aliquots of the supernatant were used for testing with the Rapid Stain Identification (RSID)^TM^-Semen and SERATEC^®^ PSA Semiquant kits.

For the RSID^TM^-Semen kit (Lot No. 051023S1), 20 µL of supernatant was diluted to 100 µL with RSID^TM^ universal buffer.

For the SERATEC^®^ PSA Semiquant kit (Lot No. 230004), 5 µL of supernatant was diluted to 120 µL with PSA Buffer Solution.

Samples were loaded onto their respective test cassettes, and the results were interpretated according to the manufacturer’s protocols. The remaining supernatant and cell pellet were subjected to DNA extraction as described below.

### 2.7. DNA Extraction

DNA extraction using the DNA IQ^TM^ Casework kit (Promega Corporation, Madison, WI, USA) on the Maxwell FSC instrument (Promega) was performed as per the manufacturer’s protocol.

For differential DNA extraction, the cell pellet and the protein supernatant were resuspended in 400 µL of lysis buffer (10 mM Tris, 10 mM EDTA, 2% (*w*/*v*) SDS, and 20 µL of Proteinase K (20 mg/mL) [16]. The mixture was incubated for 1 h at 56 °C with agitation. Intact sperm was pelleted by centrifugation, and the supernatant was collected as the epithelial fraction. The sperm pellet was washed and resuspended in 50 µL of phosphate-buffered saline to form the spermic fraction. Both epithelial and spermic fractions were subsequently subjected to Maxwell DNA extraction.

### 2.8. DNA Quantification, STR-PCR Amplification, and Amplicon Detection

Extracted DNA was quantified using the Quantifiler^®^ Trio DNA Quantification kit (Applied Biosystems, Foster City, CA, USA) on the QuanStudio^TM^ 7 Flex Real-Time PCR System (Applied Biosystems). DNA extracts were amplified in a 29-cycle reaction using the GlobalFiler^TM^ Plus kit (Applied Biosystems) on the ProFlex^TM^ PCR System (Applied Biosystems). Fluorescent amplicons were detected using the ABI PRISM^®^ 3500xL Genetic Analyzer (Applied Biosystems), with maximum injection parameters of 3 µL at 1.2 kV for 24 s. Data were analysed using QuanStudio Software version 1.7 and GeneMapper^®^ ID-X Software version 1.6, following laboratory guidelines. Percent profile recovery was calculated as the number of detected alleles relative to the expected full male profile.

## 3. Results

### 3.1. TP Does Not Affect Protein Immunoassays and DNA Processing

Based on our laboratory approach for semen examination, when the AP test yields a positive result, immunoassays may be performed to ascertain the presence of semen (Figure S1). To test whether TP could interfere with these assays, we subjected ~1 cm × 1 cm sections of the three different coloured TP samples to the reagents routinely used in RSID^TM^-Semen and Seratec^®^ PSA Semiquant assays. All TP samples produced negative results, demonstrating that TP does not produce false positives with either assay (Table 1).

**Figure 1:**
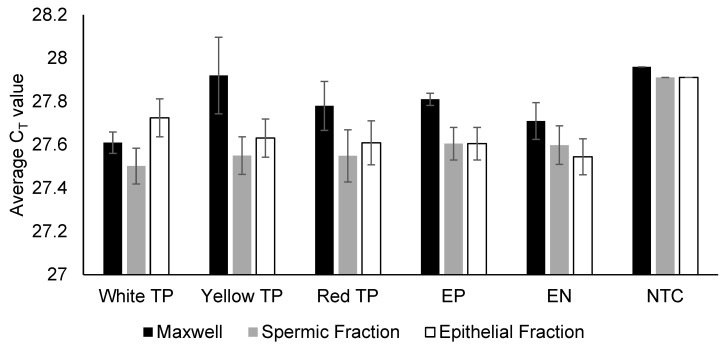
Internal PCR control (IPC) C_T_ values for TP and control samples. Sections (~1 cm × 1 cm) of the three different coloured TPs were subjected to either Maxwell (*n* = 2 for each colour) or differential (*n* = 5 for each colour) DNA extraction. Consistent IPC C_T_ values of around 27.5 (similar to those of the control samples) were obtained for all TP samples, indicating the absence of PCR inhibition, as per the manufacturer’s guidelines. EP: extraction positive; EN: extraction negative; NTC: non-template control.

Depending on the immunoassay results, the remaining material (cell pellet and protein supernatant) may either directly undergo Maxwell DNA extraction or be subjected to differential DNA extraction to separate spermic and epithelial fractions (Figure S1). DNA extraction would be followed by quantitation, STR-PCR amplification, and capillary electrophoresis detection of the fluorescent amplicons. Across both extraction methods, TP pigments did not inhibit PCR amplification, as the mean internal PCR control (IPC) C_T_ values were comparable between the TP and control samples (27.5–27.9; Figure 1). Baseline noise in electropherograms was also consistent, at ~10 relative fluorescence units (RFUs) across all dye channels (Figures S2 and S3), suggesting that TP pigment does not interfere with fluorescent amplicon detection.

### 3.2. Effect of TP Pigment on Semen Detection and DNA Recovery from Fabric

Having ascertained that the TP alone does not yield false-positive protein immunoassays or inhibit PCR amplification, we next examined whether TP pigment interferes with semen detection or DNA recovery from a mocked crime exhibit (e.g., cotton T-shirt). As expected, the TP-treated cloth samples spiked with ~5.5 µL of semen tested positive for both semenogelin and prostate-specific antigen (Table 2), confirming that the TP pigment does not interfere with detection at this level.

**Figure 2:**
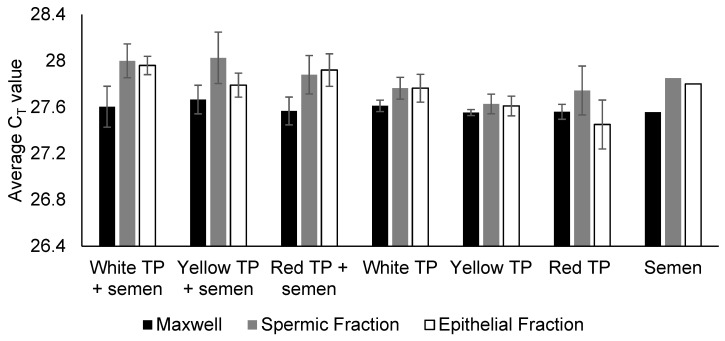
Internal PCR control (IPC) C_T_ values for TP-treated cloth samples spiked with and without semen. TP-treated cloth samples spiked with semen were extracted using Maxwell (*n* = 2 for each colour) and differential (*n* = 5 for each colour) DNA extractions. TP-treated cloth samples without addition of semen (white TP, yellow TP, red TP) were used as negative controls. Untreated cloth sample (i.e., without TP pigment) spiked with semen (labelled as "Semen") was used as positive control. Consistent IPC C_T_ values of around 27.5 were obtained for all samples, indicating the absence of PCR inhibition as per the manufacturer’s guidelines.

Quantification data from samples processed with both types of DNA extraction again showed consistent mean IPC C_T_ values (27.6–28.0) across all TP-treated cloth samples, regardless of the presence of semen (Figure 2), indicating the absence of a PCR-inhibitory effect. However, a substantial reduction in DNA yield was observed in the TP-treated cloth samples spiked with semen. Across all 36 replicates, the median yield was 1.29 ng/µL (IQR: 0.82–1.90), representing a ~70% reduction for the spermic fraction and a ~49% reduction for the epithelial fraction compared to the positive controls (Table 3). Importantly, all 36 TP-treated replicates (100%) produced lower yields than the control. Despite this reduction, complete STR profiles were recovered from all semen-spiked samples, with 100% allele recovery (no allele dropout observed), and all peaks were above the laboratory’s analytical threshold of 110 RFUs (Figure S4).

### 3.3. Transfer Paper as a Feasible Alternative Tool for Documenting AP Test Results

**Figure 3:**
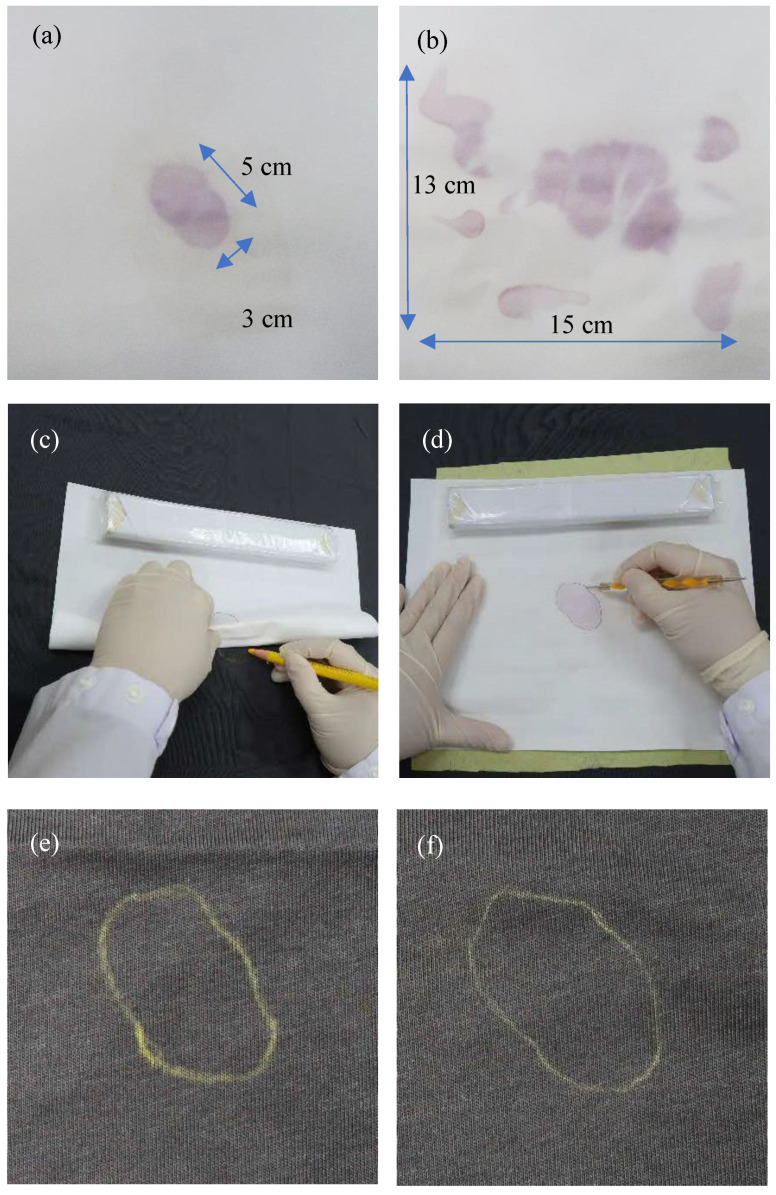
Documentation of AP test result. Positive AP test results are represented by either (**a**) a simple or (**b**) a complex purple colouration on chromatography paper. Tracing of AP test result back to a mocked crime scene item, such as a black cotton T-shirt, can be accomplished by superimposing the chromatography paper over the T-shirt and either (**c**) flipping the paper back and forth repeatedly to mark the boundary of the AP stain with a crayon or (**d**) tracing the AP stain boundary with a stylus or ballpen using TP positioned between the chromatography paper and the T-shirt. The location of the semen stain was successfully mapped with the (**e**) crayon and the (**f**) TP.

The AP test is a valuable presumptive tool for detecting semen stains in sexual crimes, where the perpetrator may deposit semen on the victim’s clothing or bedding. The AP reaction produces purple stains on chromatography paper, with patterns that vary in complexity based on the intensity and deposition mode of the semen (Figure 3a,b). Current documentation of the AP stain pattern involves flipping the chromatography paper back and forth repeatedly to mark out the AP stain boundaries with crayons onto the exhibit (Figure 3c). In the present study, using TP, boundaries could be directly replicated on the exhibit with a single or minimal tracing action (Figure 3d). Both the crayon and TP methods successfully delineated the AP stain boundaries on the exhibit (Figure 3e,f). Notably, boundaries traced with TP were fainter, owning to the indirect nature of the transfer process and the consequently lower pressure applied to the exhibit’s surface. Nevertheless, they remained sufficiently visible for stain selection.

In the final part of this study, mapping time was compared following SOP by three competent staff performing the same AP stain documentation using both crayons and TP. The mean time for TP mapping was 3.67 min compared to 20.33 min with a crayon (~5.5 fold reduction; Table 4). A Wilcoxon signed-rank test indicated no statistical significance with *n* = 3 (W = 0, *p* = 0.125), but the difference was operationally consistent and yielded a very large practical effect size (Cohen’s *d* = 9.13).

## 4. Discussion

Saral Wax-Free Transfer Paper (TP) is a type of tracing paper that facilitates the transfer of drawings or markings from one surface to another without leaving behind greasy residues [17]. While it is commercially available in five colours (white, yellow, red, blue, and graphite), we focused on white, yellow, and red due to their suitability for forensic purposes. White and yellow are ideal for tracing on dark fabric surfaces, while red is good for tracing on light fabric surfaces. While the formulation of TP is proprietary, it is understood to involve a pressure-sensitive pigment layer combined with a binder that releases TP pigment when pressure is applied. Its non-waxy, residue-free nature makes TP a suitable candidate as an alternative to crayons for documenting AP-positive areas on crime exhibits.

Our results confirm that TP does not interfere with downstream semen immunoassays or DNA processing. Specifically, there were no false-positive or false-negative reactivity RSID-Semen and Seratec PSA assays, no PCR inhibition (based on the IPC C_T_ value during real-time DNA quantification), and no elevated baseline noise during electrophoresis.

When used on semen-spiked fabric, TP pigment reduced DNA yields but did not compromise PCR and allele recovery. It should be noted that the present study intentionally employed a larger amount of TP pigment to create a stress-test condition to rigorously evaluate any potential negative effects on the forensic biology workflow. This finding is highly reassuring as it demonstrates that even direct contact with the pigment does not cause PCR inhibition and that the only risk is a yield reduction (not a failed analysis) if correct protocol is not adopted. In routine practice, only small sections (often as small as ~1 cm × 1 cm) within the AP-marked boundaries (not the boundary line) would typically be excised for DNA extraction, so TP pigment carryover into the DNA extraction process would be minimal or excluded entirely. Thus, the yield reduction observed here reflects a conservative, worst-case scenario rather than the operational reality. Even so, under these exaggerated conditions, the only observed effect was a moderate DNA yield reduction, not allele dropout. This indicates that occasional pigment transfer poses minimal risk to casework outcomes.

Beyond forensic biology workflow, potential interference with other forensic evidence types (e.g., fibres, hairs, latent prints, gunshot residues, or trace DNA) is also considered. In routine casework, AP screening is typically conducted only after these other examinations are completed, thereby minimising or eliminating the risk of interference. Where trace DNA analysis is required, swabbing would precede AP testing, ensuring that the TP-traced boundaries or crayon-marked boundaries do not affect trace recovery. Furthermore, inter-laboratory coordination ensures that distinct areas of an exhibit are assigned to the appropriate discipline. In practice, TP paper can also be cut to approximate sizes to trace only the required boundaries, further reducing any risk of secondary transfer.

In routine laboratory casework, marking AP-positive boundaries is operationally essential to relocate the AP-positive areas, particularly if the purple AP colouration fades over time or if further testing (e.g., RSID-Semen, PSA, or DNA profiling) is needed. For this reason, we did not evaluate cleaning of the TP pigment, as the marked boundary itself was intentionally retained, consistent with existing crayon-based workflows. Mapping was likewise not quantified. In routine practice, AP mapping serves as a visual guide rather than a reportable analytical result. Moreover, excisions for DNA analysis are small and taken from within the visible AP-positive region rather than the traced boundary line, making precise geometric accuracy unnecessary. Compared with crayons, AP mappings with TP were fainter and more susceptible to removal through rubbing or rough handling by staff with less experience in AP documentation. These limitations can be mitigated by re-tracing with TP or by using a thicker stylus.

Finally, the present study provides preliminary evidence that TP can substantially reduce the time required to document AP-positive areas. Mapping time with TP was approximately 5.5 fold faster than with crayons across the three competent staff. Although the small sample size limits formal inference, the consistency of the results and the large effect size (Cohen’s *d* = 9.13) support the operational significance of TP as a practical alternative.

And while TP markings are less intense and more susceptible to removal due to its wax-free formulation, its ease of use and time-saving benefits outweigh these limitations. While not groundbreaking, this study therefore underscores the potential of TP as a significant time-saving tool. At the time of this manuscript’s writing, we are unaware of any published studies detailing how AP-positive areas are documented on crime scene exhibits.

Overall, TP shows strong potential as a practical alternative to crayons for documenting AP-positive areas, with the important caveats of reduced marked intensity, reduced permanence of markings, and the proof-of-concept nature (limited fabric type, single semen source, small replicate numbers) of this study. Future work may consider expanding the evaluation to different substrates, types of stains, and allele-level DNA quality metrics.

## 5. Conclusions

This study highlights the potential of Saral Wax-Free Transfer Paper (TP) as an effective alternative to crayons for documenting AP-positive areas on cotton clothing, which is commonly encountered in sexual assault casework. The TP markings appeared fainter but remained sufficiently visible for forensic purposes. Importantly, TP did not interfere with downstream DNA processes, and although a reduction in DNA yield was observed under stress-test conditions, full STR profiles were consistently recovered. Finally, the use of TP reduced documentation time by approximately five-fold compared to the conventional crayon method, underscoring its value as a practical tool for AP mapping in forensic laboratories.

## Figures and Tables

**Table 1:** Serological testing of TP samples in the absence of semen. (RSID)^TM^-Semen and Seratec^®^ PSA Semiquant tests were conducted on different coloured TP samples.

TP Pigment Colour	RSID Result	PSA Result
White	Negative	Negative
Yellow	Negative	Negative
Red	Negative	Negative

**Table 2:** Serological testing of TP-treated cloth samples spiked with semen. RSID^TM^-Semen and Seratec^®^ PSA Semiquant tests were conducted on different coloured TP-treated cloth samples.

Semen Mixed with	RSID	PSA
White TP-treated cloth	Positive	Positive
Yellow TP-treated cloth	Positive	Positive
Red TP-treated cloth	Positive	Positive

**Table 3:** Summary of DNA yield reduction in TP-treated cloth samples spiked with semen. Data are presented as median and interquartile range (IQR) for each sample group, alongside the positive control value (i.e., untreated cloth sample spiked with semen) and calculated percentage reduction in median yield. Percentage reduction was calculated as (1 − (median/control)) × 100%.

Group	*n*	Median (ng/µL)	IQR (25th–75th Percentile)	Semen, Control Value (ng/µL)	% Reduction
All TP-treated	36	1.29	0.82–1.90	-	-
Maxwell	6	2.16	1.38–3.62	8.06	73%
Spermic fraction	15	1.67	1.16–1.90	5.51	70%
Epithelial fraction	15	0.67	0.58–0.88	1.32	49%

**Table 4:** The processing time required to document positive AP-positive stains (purple colouration on chromatography paper). Each repetition was performed by different staff to map the boundaries of AP-positive stains onto the mocked crime exhibit, i.e., a T-shirt.

	Mapping Time (Mins)	
Repetition	Crayon	Transfer Paper
1	20	4
2	23	3
3	18	4

## Data Availability

The raw data supporting the conclusions of this article will be made available by the authors upon request.

## Supplementary Materials

The following supporting information can be downloaded at https://www.mdpi.com/article/10.3390/genes16091073/s1: Figure S1: Schematic diagram illustrating the relationship between protein assays, Maxwell DNA extraction, and DNA differential extraction in our standard operating procedure; Figure S2: Average peak height of three different coloured TP swabs processed via (a) Maxwell extraction (*n* = 2 for each colour) and differential extraction, resulting in (b) spermic fraction (*n* = 5 for each colour) and (c) epithelial fraction (*n* = 5 for each colour). RFU: relative fluorescence unit; Ext neg: extraction negative; Amp neg: amplification negative; Figure S3: A representative electropherogram showing baseline noises at 80 to 260 bp in the blue dye 6-FAM channel. A TP sample processed using differential DNA extraction and its resulting spermic fraction’s electropherogram are displayed to show the background peak level. The analytical threshold was set at 1 relative fluorescence unit. From top to bottom, the figure represents white TP, yellow TP, red TP, the extraction negative control, and the amplification negative control; Figure S4: A representative electropherogram demonstrating optimal DNA profile quality. The data show complete allele recovery, good heterozygote balance, and the absence of artefacts such as a “ski-slope” effect or elevated stutter peaks.
