# Supplementary material for: Rapid Documentation of Possible Semen Stains for Forensic DNA Profiling
Source: Genes (Basel). 2025 Sep 12;16(9):1073. doi: 10.3390/genes16091073 (PMC12469308; doi:10.3390/genes16091073)
Supplement: Supplementary file 1 [file genes-16-01073-s001.zip › genes-3836777-supplementary.pdf]

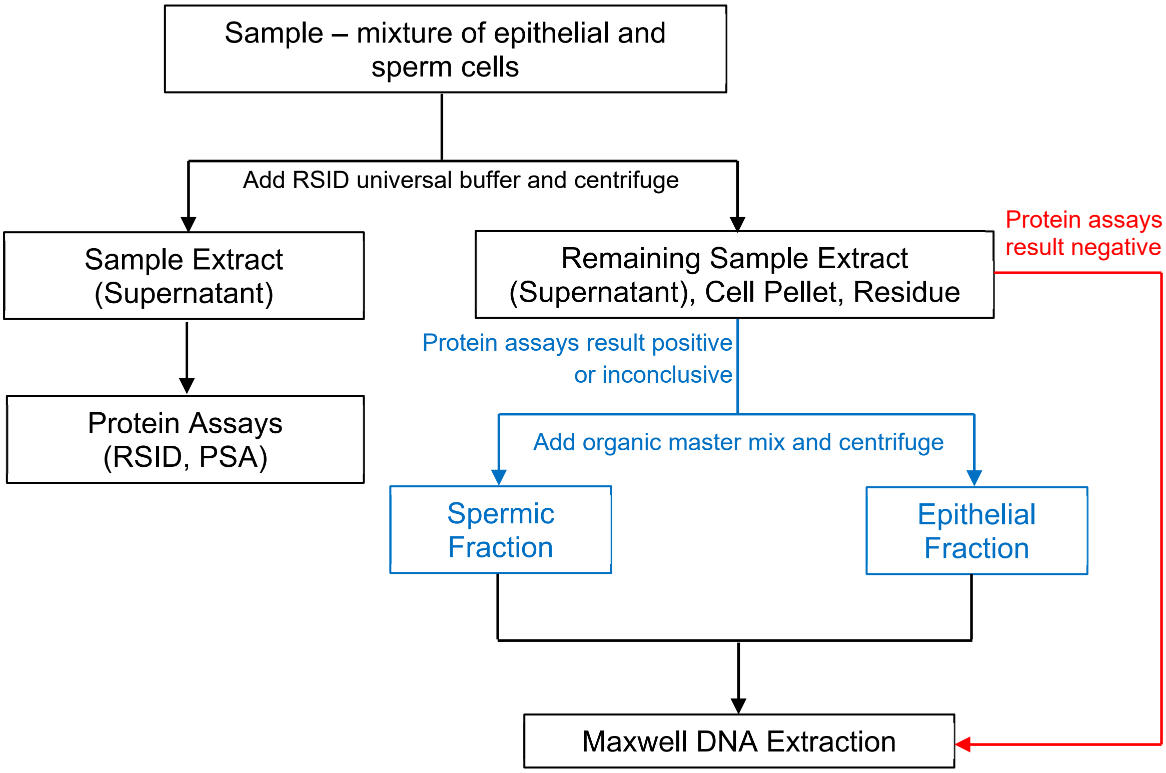

Figure S1. Schematic diagram illustrating the relationship of protein assays, Maxwell DNA extraction, and DNA differential extraction in our standard operating procedure.

(a)

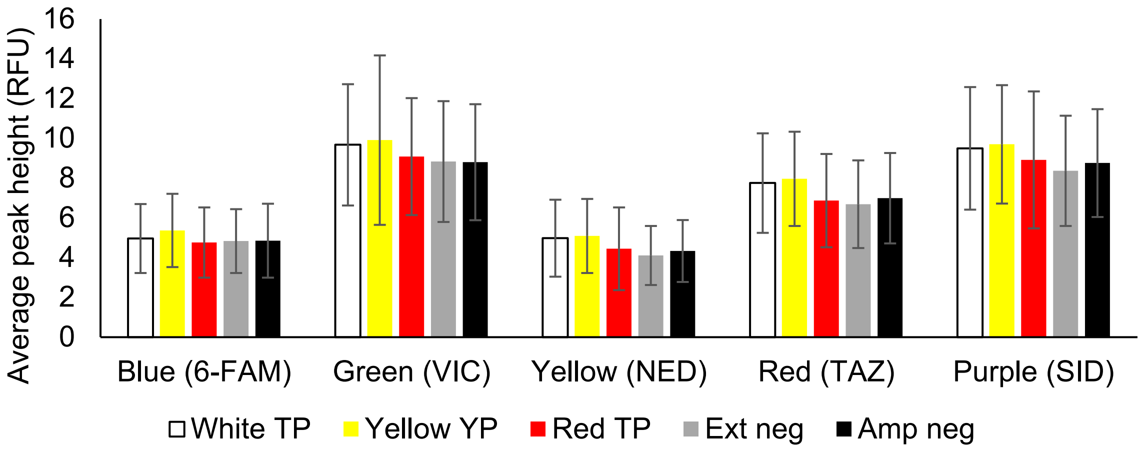

(b)

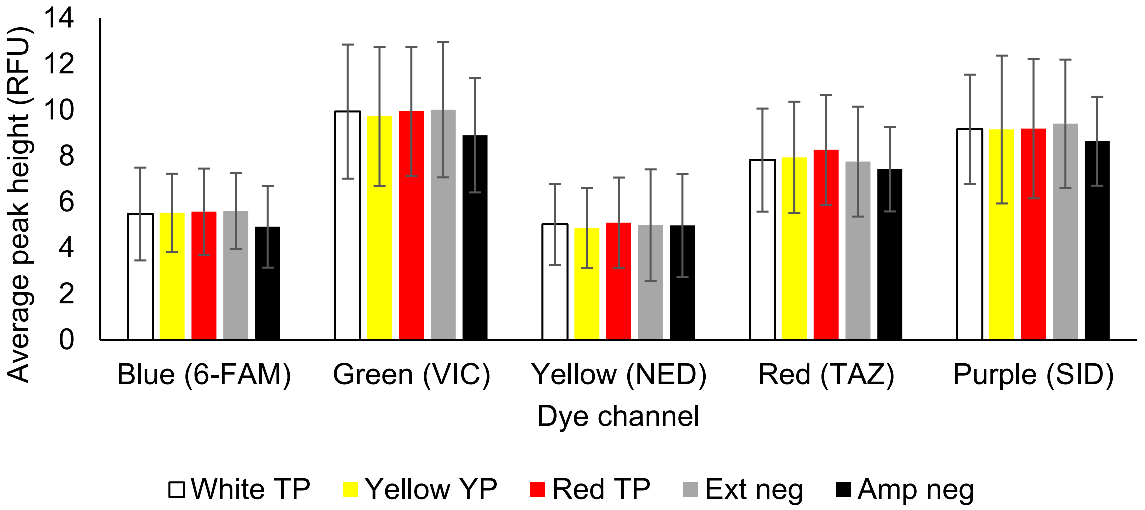

(c)

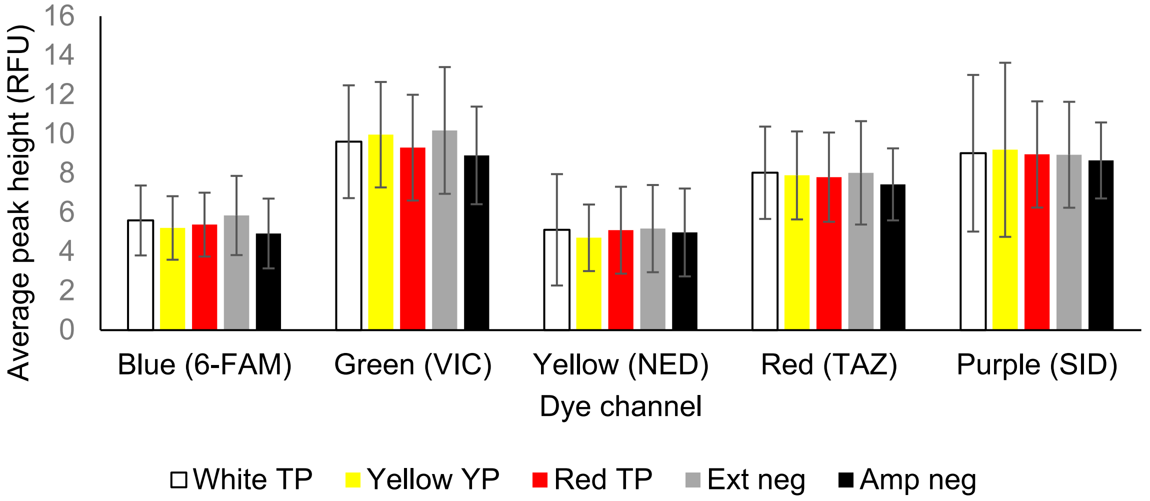

Figure S2. Average peak height of three different coloured TP swabs processed via (a) Maxwell extraction (n = 2 for each colour), and differential extraction resulting in (b) Spermic Fraction (n = 5 for each colour) and (c) Epithelial Fraction (n = 5 for each colour). RFU: relative fluorescence unit. Ext neg: extraction negative; Amp neg: amplification negative.

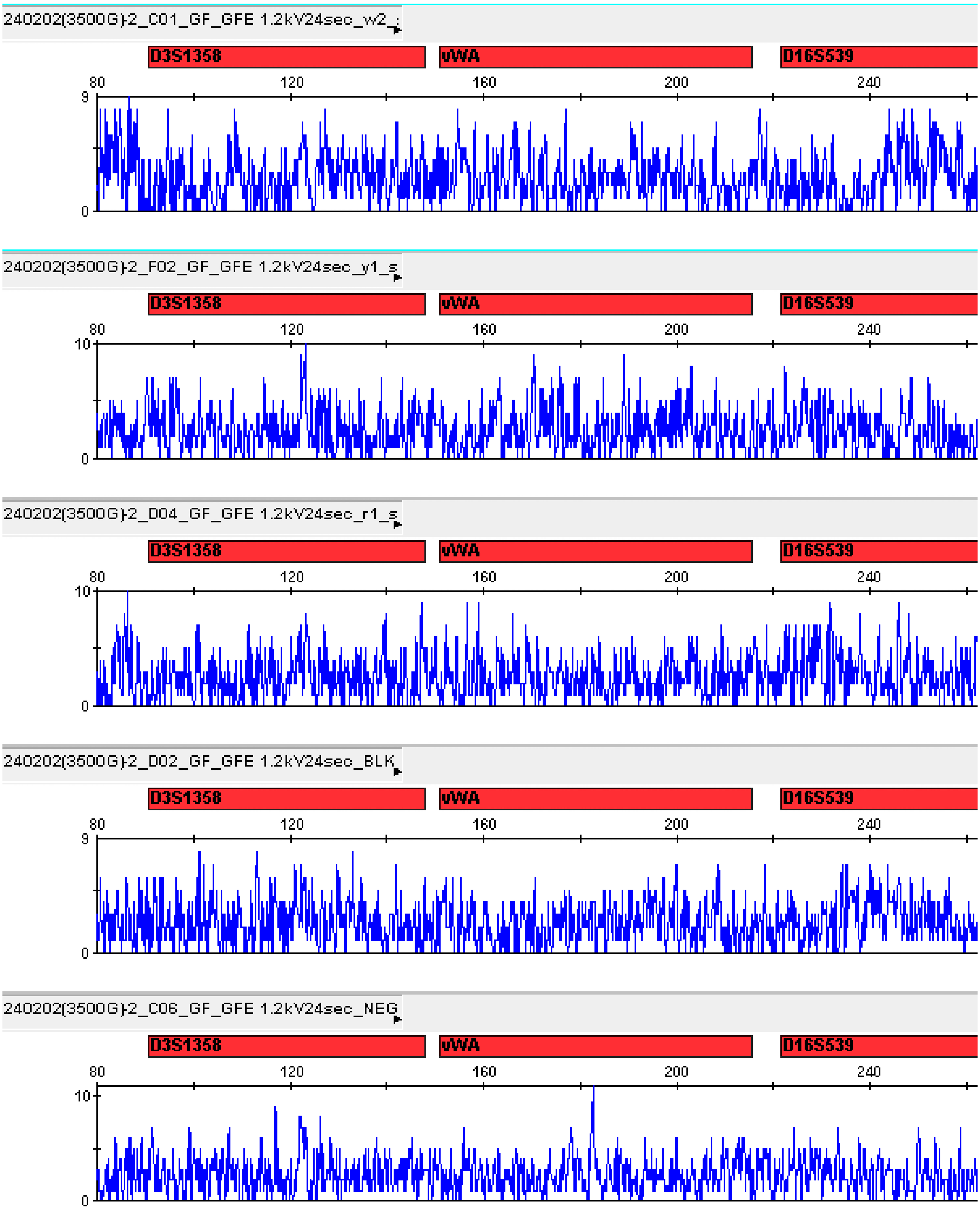

Figure S3. A representative electropherogram showing baseline noises at the 80 to 260 bp in the blue dye 6-FAM channel. TP sample processed using differential DNA extraction and its resulting spermic fraction's electropherogram was displayed to show the background peak level. The analytical threshold was set at 1 relative fluorescence unit. From top to bottom represents white TP, yellow TP, red TP, extraction negative control, and amplification negative control.

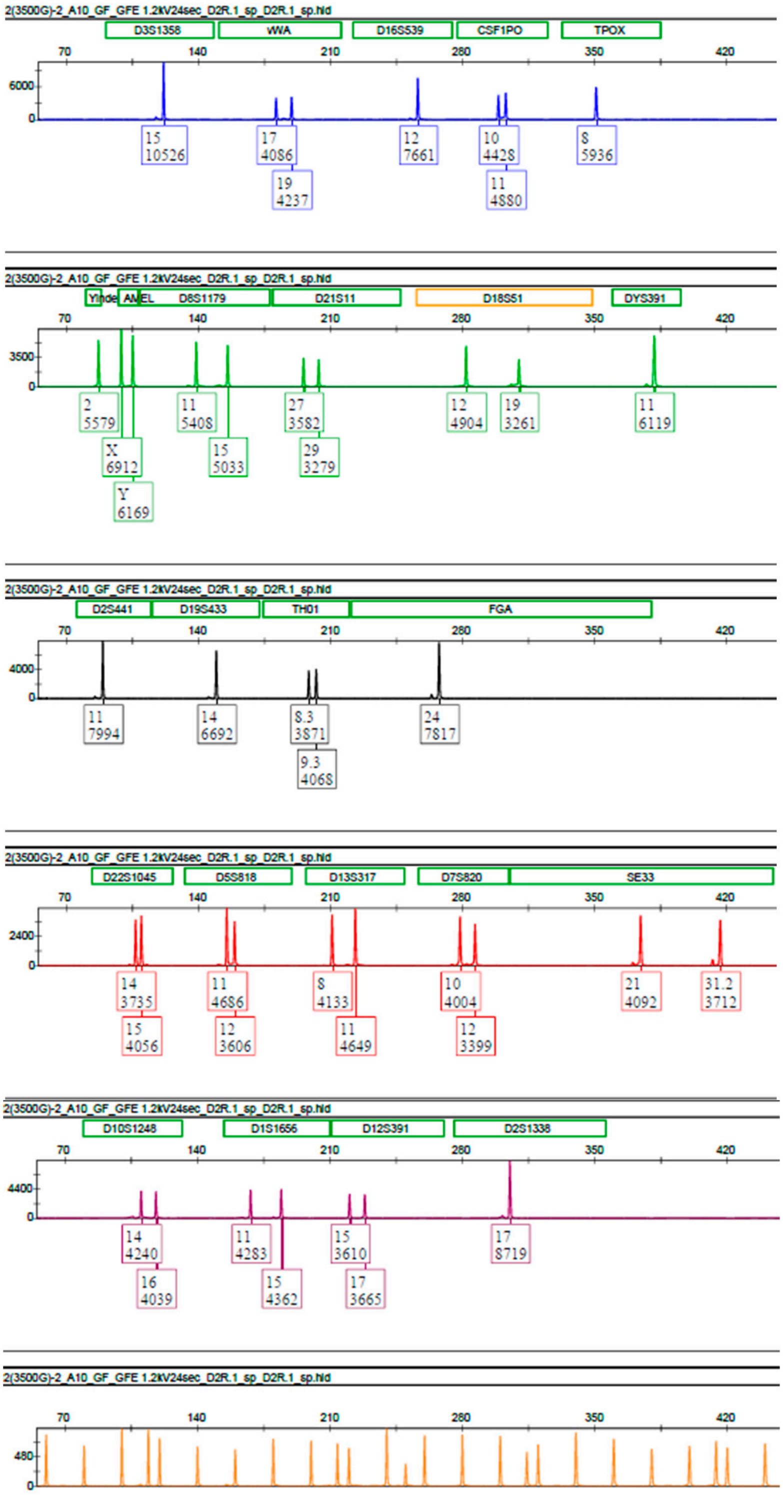

Figure S4. A representative electropherogram demonstrating optimal DNA profile quality. The data show complete allele recovery, good heterozygote balance, and an absence of artifacts such as a "ski-slope" effect or elevated stutter peaks.
